# Establishment of an overactive bladder model in mice

**DOI:** 10.1186/s12894-023-01181-1

**Published:** 2023-02-13

**Authors:** Tengfei Lv, Shan Zhong, Xiao Guo

**Affiliations:** 1 The Fourth School of Clinical Medicine, Zhejiang Chinese Medical University, Hangzhou, China; 2 The Department of Urology Surgery, The Second Affiliated Hospital of Jiaxing University, Jiaxing, China; 3 The Department of Urology, Huashan Hospital Affiliated to Fudan University, Shanghai, China

**Keywords:** Overactive bladder, Partial bladder outlet obstruction, Mouse model

## Abstract

**Background:**

Overactive bladder (OAB) is a syndrome characterized by symptoms of urinary urgency, often accompanied by frequent urination and nocturia or urge incontinence.

**Methods:**

Twenty female ICR mice were randomly divided into pBOO (partial bladder outlet obstruction) and control groups. The mouse OAB model was constructed by ligating the bladder outlet. Eight weeks after the operation, the methods of voiding spot on paper (VSOP), isolated detrusor muscle, and HE staining were used for analysis and research.

**Results:**

After the operation, two mice in the experimental and one in control died, and one in the control groups had an abnormal bladder size, so it was excluded from the statistical analysis. Eight weeks after the operation, there was an insignificant difference (*P* = 0.15) in the body weight of mice in the pBOO (26.54 ± 2.62 g) and the control group (24.84 ± 1.76 g). The number of urinations in 12 h was significantly higher (*P* < 0.001) in the pBOO (7.63 ± 1.19) than in the control group (4.13 ± 0.99). Also, the 12-h urine volume of pBOO (1491.23 ± 94.72 μL) was significantly greater (*P* = 0.006) than that of the control group (1344.86 ± 88.17 μL). The isolated bladder of the pBOO mice was significantly heavier than that in the control group (53.16 ± 1.79 mg vs. 24.54 ± 1.80 mg, *P* < 0.001), the horizontal and vertical length of the bladder in pBOO group were larger than those in the control group (*P* < 0.001). The detrusor thickness of pBOO group (357.50 ± 11.88 µm) was significantly thicker than that of control group (258.52 ± 17.22 µm, *P* < 0.001), and the isolated muscle strip was more sensitive to carbachol stimulation. According to HE staining, the bladder wall of the pBOO mice was significantly thickened.

**Conclusions:**

A pBOO-mediated mouse OAB model was successfully established by ligating the bladder outlet.

## Background

Overactive bladder (OAB) is defined by the International Continence Society (ICS) as "a syndrome characterized by urinary urgency, often accompanied by increased frequency and nocturia, with or without urge incontinence, excluding symptoms due to acute urinary tract infection or other forms of localized disease of the bladder and urethra" [1]. As a chronic disease, OAB seriously affects patients’ quality of life [2, 3], and the incidence of OAB also increases with age [4, 5]. However, the pathogenesis of OAB is still unclear. Two theories have been proposed, the myogenic and the neurogenic theory. The myogenic theory believes that bladder outlet obstruction (BOO) increases intravesical pressure, and bladder distension leads to partial denervation of bladder smooth muscle, resulting in various functional smooth muscle changes [6]. The neurogenic theory believes that due to the damage to the central inhibitory pathways in the brain and spinal cord or the sensitivity of peripheral nerve afferents in the bladder, the original voiding reflex cannot be inhibited, resulting in detrusor overactivity [7]. Many treatments alleviate the symptoms, but they do not cure OAB [8–10]. Studies using animal models are particularly important to better understand OAB pathogenesis and its treatments. The use of animals to establish an OAB model is relatively common but not the use of mice; thus, this study established a disease model of OAB by constructing a partial BOO (pBOO) in mice.

## Methods

### Model establishment

28-week-old female ICR mice weighing 25 ± 2 g were randomly divided into the pBOO group (n = 10) and the control group (n = 10). The mice had free access to food and water and were housed in 24 ± 2 °C with 50–70% humidity and a 12 h light/dark cycle. The mice were deprived of water for 4 h before surgery and were anesthetized by intraperitoneal injection of sodium phenobarbital (33 mg/kg), then fixed in a supine position on a temperature-controlled operating table. The abdomen was sterilized, and the abdominal cavity was opened at the midline of the lower abdomen and 1.5 cm above the pubic bone to expose the bladder fully. The bladder was pulled to the head to expose the bladder neck area, and the tissues on both sides of the bladder neck were slightly dissociated to identify the bilateral ureters. To establish the pBOO, the tissues around the urethra were fully dissected, and a 6–0 Vicryl silk thread passed through the back of the bladder neck urethra. A 22G needle was placed beside the urethra, and the urethra and the needle were tied together before the needle was removed. Compression was used to stop bleeding, and the incision was sutured in layers and disinfected. In the control group, only the bladder neck was exposed freely without ligation, and the incision was closed layer by layer. After the operation, the mice were put into recovery cages and treated with analgesia (buprenorphine 0.1 mg/kg, twice a day) and antibiotics (intramuscular injection of penicillin in the lower limbs for three consecutive days). After the mice were observed to move freely, they were returned to the normal rearing cage.

### Metabolic cage study

The bladder function of the mice was assessed 8 weeks after surgery using voiding spot on paper (VSOP) to record and analyze the amount and frequency of urination [11, 12]. The urination traces of each mouse were captured by placing a Whatman filter paper on the bottom of the cage, which was moved under a waterproof wire mesh at a speed of 5 cm/h. The Whatman filter paper was then visualized with UV light, and image analysis was performed using ImageJ software to quantify the mouse urine output [13, 14]. Before the start of the experiment, the mice were put into the metabolic cages for 1 day and then placed in the metabolic cages alone. During this period, they fasted and were free to drink water for 12 h (the fixed daytime time was 7:00 a.m. to 7 p.m.), and the urination traces were captured at the bottom of the cage [15].

### Bladder collection

After the metabolic cage experiment, the mice were weighed and sacrificed. The bladder was dissociated immediately and weighed immediately after all the urine was squeezed out. Samples were placed in a Ca^2+^-free solution, and the surrounding tissues were trimmed; after dissection along the middle of the bladder, the mucosa and lamina propria were carefully removed under the microscope, and the maximum horizontal and vertical length of the bladder were measured. The detrusor muscle strips were cut along the longitudinal axis to a size of 1 mm × 5 mm. The detrusor muscle strips were fixed in the water tank of Krebs solution and then heated at 37 °C for stimulation experiments with different concentrations of carbachol (cch). After the tension transducer was converted, a multi-channel physiological collector was used to analyze the tension curve. The bladder detrusor tissue was fixed, dehydrated, embedded, and sectioned, followed by HE staining, and the changes in the bladder tissue were observed under a microscope.

### Statistics

SPSS software was used for statistical analysis, and all data were expressed as mean ± standard deviation (mean ± SD). The data were analyzed by t-tests, and *P* < 0.05 was considered statistically significant.

## Results

### Animal model

One pBOO mouse died during the operation, one mouse died 2 weeks after the operation, and eight mice survived. One control mouse died the 1st week after the operation, and nine mice survived. During the autopsy, one control mouse had a significantly enlarged bladder, which was considered to be the cause of the sham operation failure, so the data were excluded from the statistical analysis, so the experimental and the control groups contained eight mice.

### Metabolic cage study

Eight weeks after the operation, there was an insignificant difference in the body weight of the mice in pBOO and the control groups (*P* = 0.15). The urination behavior of the mice was observed in the metabolic cage showing 12-h urination frequency (*P* < 0.001) and 12-h urination volume (*P* = 0.006) of pBOO mice were significantly greater than that in the control group (Table 1).

**Table 1:** Results of mice in pBOO and control groups at 8 weeks after operation

Items	pBOO group (n = 8)	Control group (n = 8)	*P* value
12-h urination frequency	7.63 ± 1.19	4.13 ± 0.99	< 0.001
12-h urination volume (µL)	1491.23 ± 94.72	1344.86 ± 88.17	0.006
Body weight of the mice (g)	26.54 ± 2.62	24.84 ± 1.76	0.15
Bladder weight (mg)	53.16 ± 1.79	24.54 ± 1.80	< 0.001
Bladder horizontal length (mm)	5.76 ± 0.32	3.01 ± 0.22	< 0.001
Bladder vertical length (mm)	4.94 ± 0.23	3.56 ± 0.37	< 0.001
Detrusor thickness (µm)	357.50 ± 11.88	258.52 ± 17.22	< 0.001
Detrusor strips contraction to carbachol (3 × 10^−8^ M)	170.75 ± 9.16	1.25 ± 0.46	< 0.001
Detrusor strips contraction to carbachol (1 × 10^−7^ M)	213.00 ± 12.66	79.38 ± 7.98	< 0.001
Detrusor strips contraction to carbachol (3 × 10^−7^ M)	317.63 ± 27.99	180.51 ± 11.36	< 0.001

### Bladder collection

**Fig. 1:**
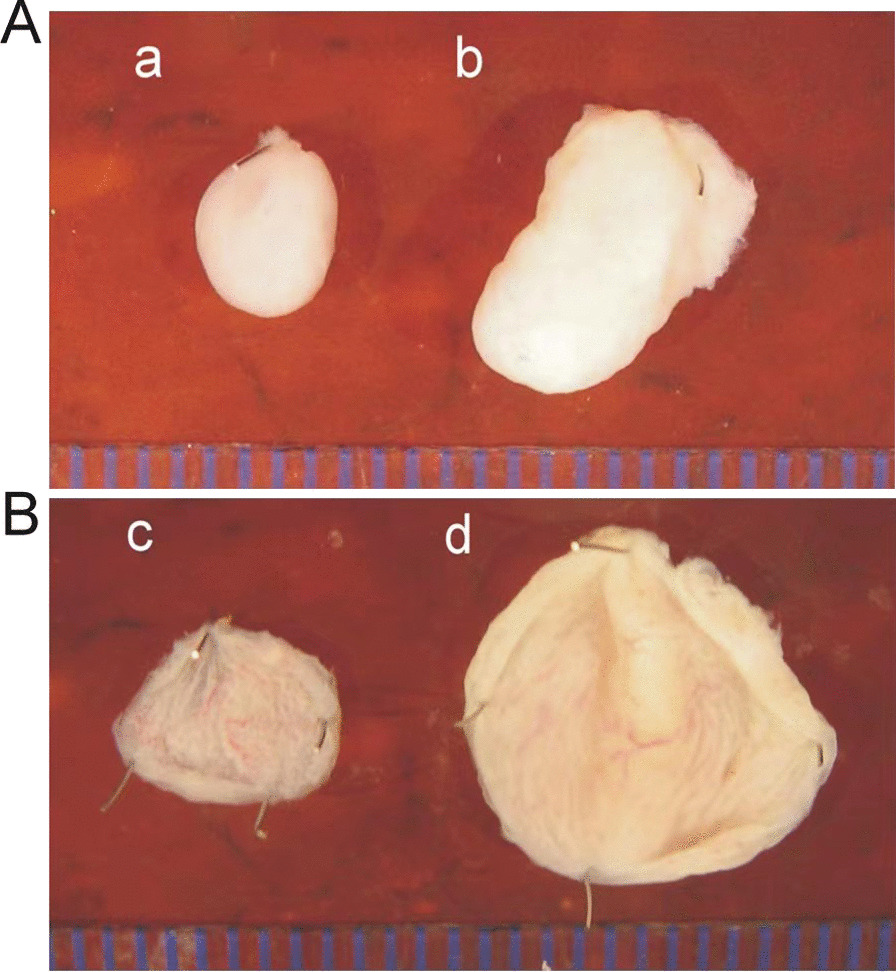
**Aa, Bc** The bladder of the control group; **Ab, Bd** the bladder of the experimental group was significantly distended, and the bladder wall was hypertrophied

**Fig. 2:**
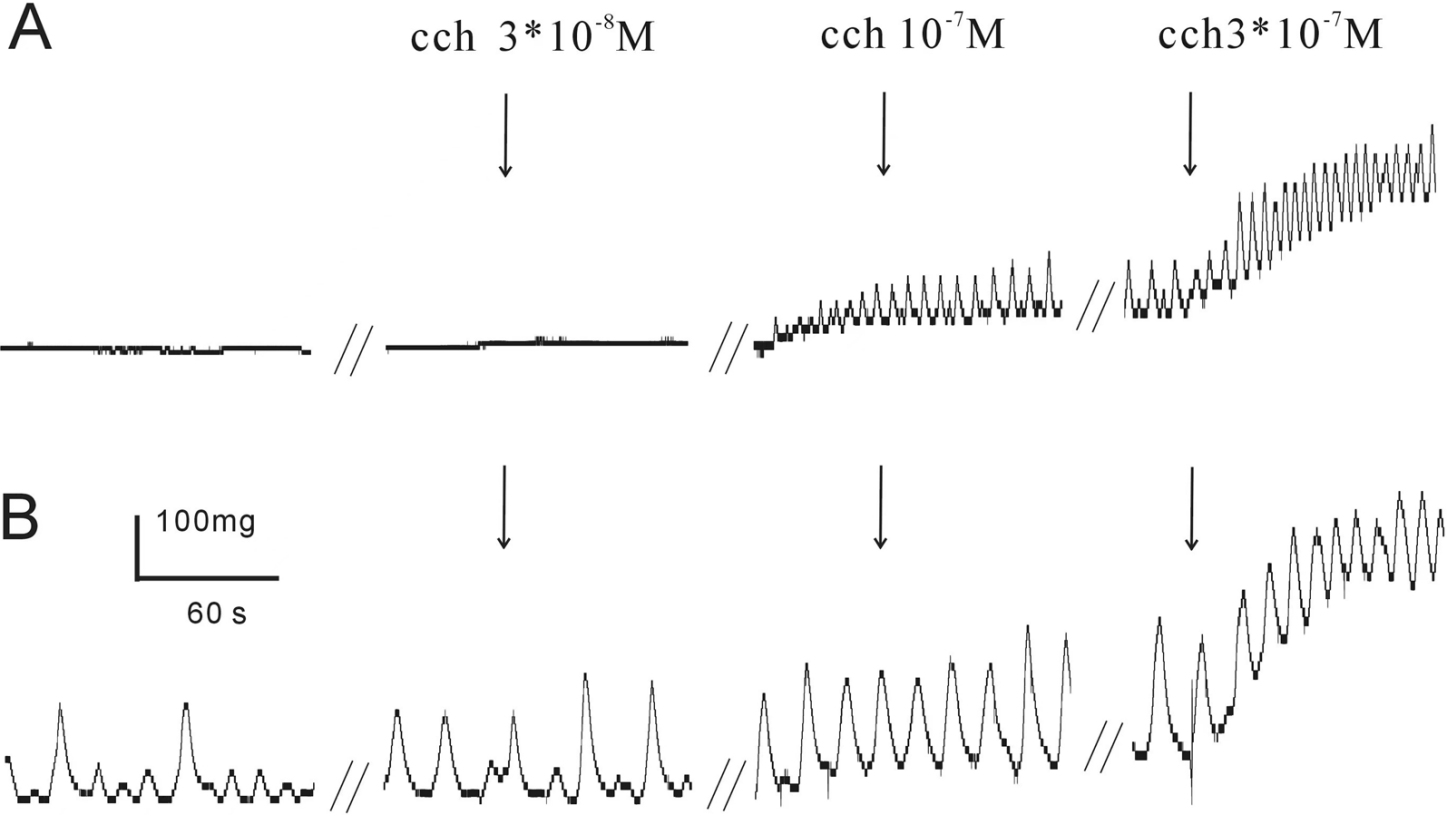
Spontaneous contraction of the bladder detrusor strips and response to M receptor agonist carbachol (cch). **A** Bladder detrusor muscle of control mice; **B** the bladder detrusor muscle of pBOO mice contracted spontaneously and was significantly sensitive to cch stimulation

**Fig. 3:**
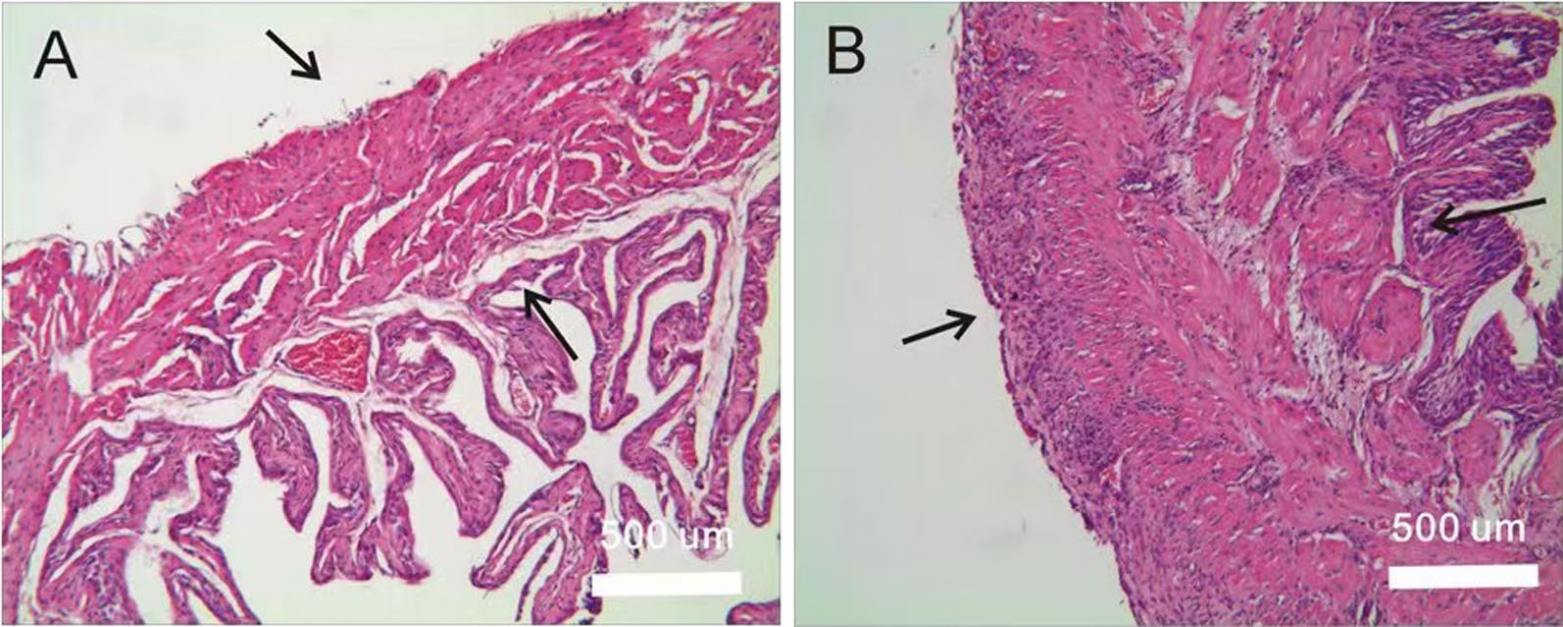
Bladder wall HE staining. **A** The bladder wall of the control mice; **B** the detrusor layer of pBOO mice displaying hyperplasia and hypertrophy

The volume of the isolated bladders of pBOO mice was significantly larger than that in the control group, as demonstrated in Fig. 1. Furthermore, compared to the control bladders, pBOO mice had more tissues and fat around the bladder. The horizontal and vertical length of the bladder in pBOO group were larger than those in the control group (*P* < 0.001, Table 1). When the isolated bladder was weighed, pBOO group was significantly heavier (*P* < 0.001) than the control group. Also, the detrusor thickness of pBOO group (357.50 ± 11.88 µm) was significantly thicker than that of control group (258.52 ± 17.22 µm, *P* < 0.001). (Table 1). The detrusor muscle strips in the pBOO group were more sensitive to carbachol stimulation, and the bladder muscle strips in the pBOO group had spontaneous contractions, as displayed in Fig. 2 and Table 1. HE staining revealed that the bladder muscle layer of the pBOO mice was significantly thicker than that in the control group, as illustrated in Fig. 3.

## Discussion

The overactive bladder is an important part of urological research. Although cell biology and molecular biology have developed rapidly [16, 17], the use of animal models to observe the biological activities and overall status of the body is necessary [18]. Rodent models are important for studying bladder physiology and pathophysiology [19]. Currently, the most commonly used surgical procedure for establishing an overactive bladder model is the creation of a partial bladder outlet obstruction [3].

Morphologically, the mouse bladder structure is similar to humans [22], so ICR mice were used to establish the model. Since mice are more fragile than rats, attention should be paid to gentle movements during surgery and close care after surgery. Nonetheless, one pBOO mouse died during the operation, one died after the operation, and one mouse in the sham operation group also died after the operation.

Due to the small size of mice, the volume of each urination is very small and difficult to record, so evaluating urination in mice is challenging. Although cystometry is often used to assess bladder function in mice, multiple potential pitfalls of cystometry have been demonstrated in previous studies [19]. With the development of plaque assays on paper, the assessment of urination in mice has also become easier and more convenient [11]. The analysis of plaques on paper of mice in metabolic cages found that the urination frequency of pBOO mice was higher than that in the control group, indicating that this overactive bladder mouse model was successfully established [13, 20].

One mouse in the sham-operated group had a higher urination frequency, and its bladder was also significantly enlarged after dissection, which may be due to the dissection of the bladder and surrounding tissues of the urinary tract through mid-abdominal incision damage to the bladder. Inflammation during the healing of abdominal incisions may also contribute to bladder dysfunction. In general, the bladder of the pBOO mice is significantly expanded, and the bladder wall is hypertrophic.

In this study, the detrusor muscle of pBOO mice contracted irregularly and spontaneously, and the response to carbachol was significantly enhanced, indicating that bladder outlet obstruction increased the excitability of the detrusor muscle. There was obvious hyperplasia and hypertrophy of the muscle layer in the bladder tissue sections of pBOO mice, confirming the establishment of the animal model [21]. The badders of pBOO mice were larger than those of the control group, indicating that bladder outlet obstruction will compensate for the bladder, resulting in hyperplasia and hypertrophy of the bladder muscle layer, which is consistent with the results of previous studies [22, 23].

This study has some limitations. The animals used in our study were all female mice, so it is unknown whether this model can be adapted to male mice. Postoperative care needs to be strengthened to avoid mice dying after surgery. This metabolic cage study only observed and recorded the urination behavior of mice during the day, and it is uncertain whether it influences the urination behavior of mice at night. The overall duration of this experiment was not sufficiently long to study the long-term partial obstruction of the bladder outlet in mice. Future studies should prolong the survival time of mice after surgery and analyze the urination behavior of mice more frequently to better describe the changes in bladder function and morphology after partial bladder outlet obstruction. The effects of different drugs on the treatment of overactive bladder should also be investigated. Based on this model, multidisciplinary techniques such as electrophysiology, immunohistochemistry, molecular biology, and biomechanics can also be used to explore the mechanism of detrusor cell remodeling in the overactive bladder to further reveal the pathogenesis of OAB.

## Conclusion

This study established a pBOO-mediated mouse OAB model by ligating the bladder outlet. Evaluation by the VSOP method, in vitro bladder test, and HE staining showed that this model is an appropriate mouse OAB model.

## Data Availability

The dataset supporting the conclusions of this article is included within the article.
